# Biomarker in Hepatocellular Carcinoma

**DOI:** 10.1007/s13193-023-01858-x

**Published:** 2024-01-02

**Authors:** Pooja Basthi Mohan, Rajiv Lochan, Shiran Shetty

**Affiliations:** 1 Department of Gastroenterology and Hepatology, Kasturba Medical College, Manipal Academy of Higher Education, Manipal, 576104 Karnataka India; 2 Lead Consultant Surgeon - HPB and Liver transplantation Surgery, Manipal Hospital, Bengaluru, 560017 Karnataka India

**Keywords:** Hepatocellular carcinoma, Alpha-fetoprotein, Biomarker, HCC, NAFLD

## Abstract

Liver cancer is one of the most prevalent types of cancer and a major contributor to the socioeconomic burden worldwide. The pathogenesis of hepatocellular carcinoma (HCC) is contributed by various etiological factors like virus infection, excessive alcohol consumption, exposure to toxins, or metabolic disorders. Majority of patients are diagnosed with late-stage HCC, which restricts its management to only palliative care. HCC, if diagnosed early, increases the survival and quality of life. Currently available biomarker (alpha-fetoproteins) have several limitations, that impede the early diagnosis and staging of cancer. This warrants the continous search in pursuit of a novel biomarker. Several research works in diverse areas have contributed to the identification of various novel biomarkers that have shown multifaceted application in early disease diagnosis, which further aid in targeted and effective therapy that can prevent cancer progression. This improves the overall health status of the patient along with significant reduction in caretaker’s burden. With the aid of novel technologies, several biomarkers have been investigated and validated in mutliple preliminary research works. Therefore in this review, we have outlined various novel biomarkers that showed promising outcomes in their trials and we have highlighted the developing areas that act as game changers in cancer diagnosis and management.

## Introduction

Hepatocellular carcinoma (HCC) is one of the main causes of cancer-related deaths worldwide. Around 20% of individuals with HCC don’t have more than 1 year survival duration post diagnosis. HCC accounts for an incidence of 800,000 cases per year with the global death rate 8.2%. Reduced prognosis rate in HCC patients are caused by several issues, which include ineffective early detection methods, a lack of curative therapies for patients who are detected at a later stage, inconsistent curative therapy application in clinical practice, and competing mortality risks from comorbid liver disease. Ideally, screening would enable the pick-up of the most widely used classification Barcelona Clinic Liver Cancer (BCLC) algorithm for lesions at early stages [3]. HCC is usually diagnosed during the later stages of the disease as the tumor is often asymptomatic [4].

In developing countries like India, information on HCC is inadequate. The incidence of HCC in cirrhotics in India was observed to be 1.6% per year of all the cirrhosis patients [5]. In 2009, the Indian Council of Medical Research cancer registries documented 25,000 cases of HCC [6], which indicates that HCC frequency either is increasing or is being diagnosed more often.

The pathogenesis of HCC is complex; usually, chronic liver damage caused by hepatitis virus (hepatitis B virus (HBV) and hepatitis C virus (HCV)) creates an environment where HCC can grow, suggesting that the disease’s pathogenesis is immune-mediated. Alcohol consumption has been associated with an increased risk of several malignancies; alcohol causes liver damage and leads to an increased risk of HCC through direct (genotoxic) and indirect (cirrhosis) mechanisms [7].

In patients with nonalcoholic steatohepatitis (NASH), the HCC pathogenesis follows a unique pattern that includes chronic hepatitis, necroinflammation, and a complex metabolic disease. Oxidative stress and DNA damage serve as prerequisite conditions for the development of HCC malignancy in nonalcoholic fatty liver disease (NAFLD) patients [8]. Presently, the worldwide NAFLD burden is increasing at an alarming rate. The study by Musunuri et al. [9] showed that HCC due to NAFLD appears to be overtaking HBV as the common etiology of HCC. The epidemiological study by Acharya et al. [6] analyzed four large case series reports of various tertiary care centers in India and found no details on HCC burden data.

Potentially curative treatments, adopted surgery (liver transplantation), and liver resection are currently dependent on early diagnosis, which is attained through surveillance of high-risk patients using routine ultrasonography, with or without measurement of the tumor marker [10]. There is a need for a persistent concerted effort to produce data on the correlation between the existing candidate biomarkers and novel biomarkers that are generated from the recently available research. The World Health Organization states a biomarker as a substance or structure that can be measured in the body to predict the incidence of disease. Alpha-fetoprotein (AFP) is the most frequently used biomarker for HCC throughout the past several years [11]. After birth, serum AFP levels frequently drop and attain levels in adulthood. However, AFP level rises yet again in HCC [12]. Morever, these trends have shortcomings and neither the European nor the American guidelines included the quantification of serum AFP for HCC screening and diagnosis due to its poor sensitivity and specificity [13, 14].

Even though biomarker-based cancer screening is still a rare paradigm, detection of HCC in the early stage using imaging and biomarkers might dramatically improve patient outcomes. The combination of various omics technologies could deliver more accurate detection for HCC, particularly in the initial stage [15]. Apart from the biomarker that is currently present in clinical settings, several other molecules are under investigation. In this review, we have provided an overview of currently available and ongoing research studies on HCC biomarkers, along with cutting-edge techniques that might aid in early detection and diagnosis of HCC.

## Biomarker in Clinical Practice

### AFP

The most widely utilized biomarker for HCC surveillance is AFP. It is a 70-kD glycoprotein, which is structurally similar to albumin and is synthesized by the fetal liver cells during pregnancy. AFP level declines rapidly after birth. However, if the liver is injured or during liver cancer, it can again drastically rise in the patient’s serum. It was first identified as a supportive biomarker for HCC diagnosis over five decades ago in animal models [16]. In a case–control study by Choi et al., high AFP levels were detected 6 months before the diagnosis of HCC, indicating that it could be useful for HCC diagnosis [17]. A meta-analysis by Xu et al. showed that AFP assays for the diagnosis of HCC had a combined 51.9% sensitivity and 94% specificity and with an area under the curve value of 0.81 [18].

Despite being introduced as an HCC screening tool, AFP’s efficacy has been questioned and it is not advised to use this test alone for screening. The European Association for the Study of the Liver suggests liver ultrasonography over AFP for HCC surveillance. Nevertheless, AFP is a useful adjunct diagnostic biomarker for the identification and monitoring of HCC. The results of the meta-analysis of 13 studies conducted by Singal et al. defined no significant alterations in sensitivities for HCC diagnosis at the initial stages with ultrasound alone and when combined with AFP [19]. In contrast, the results of the recent meta-analysis conducted by Tzartzeva et al. of 32 studies showed that the ultrasound with AFP had significantly high sensitivity for early diagnosis of HCC with a *P* value of 0.002 [20].

AFP-L3 is one malignant tumor-specific isoform of AFP. It is more specific than AFP and is derived from cancer cells. AFP-L3 helps in identifying patients with a high risk of HCC who need constant monitoring and has been accepted by the FDA for evaluating the risk of HCC. Several studies show that AFP-L3 displays a better specificity but lower sensitivity for HCC detection at an early stage compared to AFP [21]. A retrospective study conducted in Japan by Shiraki et al. [22] demonstrated that 95% and 71% of patients had positive values of AFP-L3 at 3 and 6 months before diagnosis of HCC. A study comparing AFP, AFP-L3, and DCP for the diagnosis of HCC nodule (< 5 cm) showed AUROCs for all these biomarkers to be above 0.65 [23].

### Des-Gamma-Carboxy-Prothrombin (DCP)

DCP is a vitamin K deletion II by a non-functional prothrombin molecule. It is defined as both a paracrine factor that engages in the interaction between vascular endothelial cells and an autologous growth factor that fosters the formation of HCC. A study by Nakamura et al. conducted in Japan showed that the accuracy of DCP was superior to AFP, particularly for large tumors [24]. DCP is also a potential predictive factor for HCC recurrence following treatment. In 2014, a UK-based prospective single-center study on 670 chronic liver disease (CLD) patients was carried out to identify the HCC biomarkers. Scoring system was adapted for HCC diagnosis called “GALAD” score which comprised of variables like age, gender, AFP-L3, and DCP. The study outcome displayed excellent performances in diagnosing. The GALAD score was then validated in a large worldwide cohort, and it subsequently had the ability to differentiate HCC from CLD [25]. A study from Japan and Germany showed similar results on the GALAD score in HCC detection among NASH patients [26].

Despite the remarkable performances, GALAD scoring model incorporation in real-time clinical practice is slow, and has not been officially endorsed by the major liver societies [27]. The FDA has accepted DCP as a biomarker for predicting the risk of HCC [28]. Notably, according to Chinese and Japanese guidelines, AFP, DCP, and AFP-L3 have been recommended for clinical practice. A recent study on combination of biomarkers AFP, DCP, and AFP-L3 did not raise its performance in HCC detection at the initial stage when compared to AFP and AFP-L3 alone [29].

### Glypican 3 (GPC3)

GPC3 is a proteoglycan made of heparan sulfate and is closely related to the growth of tumors and has a significant function in cell proliferation and differentiation. It is rarely expressed in normal hepatocytes and overexpressed in HCC tissues [30, 31]. In the recent findings, high GPC3 expressions were found in HCC tissues [32]. A meta-analysis by Li et al. analyzing the prognostic ability of GPC3 in HCC management found that its overexpression is associated with poor prognosis [33]. GPC3 has been used as a target for molecular imaging and therapeutic intervention in HCC. For the early detection of HCC, research is still being done employing GPC3-targeted magnetic resonance imaging, positron emission tomography, and near-infrared imaging. Additionally, serum GPC3 can also help in differentiating between patients with early liver cancer from those without HCC [34]. However, in HCC diagnosis, the accuracy level for the detection of GPC3 is highly effective and shows great results in tissue biopsies. To determine whether serum GPC3 can be used as a non-invasive diagnostic marker for HCC, more research should be done.

## Fallacies of the Present Biomarker

AFP is the most common biomarker used for the screening of HCC. However, it is also used in the diagnostic assessment of other hepatic and non-hepatic conditions. The study has reported that persistently elevated AFP is present in patients with non-malignant conditions, which can confound the ability of the AFP to primarily diagnose HCC [36, 37]. According to reports, AFP is elevated in certain hereditary disorders; HPAFP (hereditary persistence of AFP) is a rare autosomal dominant state in the literature with 20 reported cases, which reported to have AFP levels up to 1500 ng/mL [35, 36]. Another study conducted in 1984 by Greenberg et al. reported that a 38-year-old woman from a Scottish family was noted to have persistently elevated AFP during post-partum [37]. Present reproaches on the use of AFP mainly target its unsatisfactory sensitivity and specificity for the detection of HCC at the early stage if used alone. Patients with cirrhosis, active hepatitis, increased blood alanine aminotransferase, or non-HCC malignancies may have elevated AFP levels. Moreover, in some cases, AFP levels remain normal in 15–30% of patients with CLD leading to high negative rates [36]. There are continuous efforts to look for new blood-based HCC biomarkers due to the limitations of AFP.

## Novel Biomarkers for Hepatocellular Carcinoma

The advance in cancer biology has improved significantly because of the advancement of detecting technology. Many biotechnological methods such as chemiluminescence immunoassay, ELISA, immunosensor, and liquid biopsy help in better diagnosis of HCC. With the introduction of next-generation sequencing, our ability to examine the cancer genome has increased, consequently enhancing the identification of diverse range of HCC biomarkers based on the pathogenesis of HCC. The recently identified novel biomarkers are described in the following section.

### Genetic Biomarkers

The biology of liver cancer is heterogeneous. It involves various genetic alterations in a single patient, which is the reason for the limited performance of biomarkers [38]. Genomic analyses help in better characterization of a tumor, which further aid in the treatment optimization of HCC patients [39]. In a large-scale investigation of HCC-specific mutations, deletions, or epigenetics, alterations occur in at least one of 31 different genes [38]. HCC screen, a type of liquid biopsy assay, was developed by Duan et al. that could identify HCC in asymptomatic HBsAg-seropositive patients [40]. Another study on exosome sequencing showed the correlation between mutation in CTNNB1 gene and alcoholic HCC. There are several other proto-genes that undergo mutation in HCC such as CDKN2A [41].

Several types of RNAs present in the serum have gained the attention in the HCC diagnosis. microRNAs (miRNA) are chemically unstable non-coding RNAs consisting of approximately 22 nucleotides [42]. The progression of cancer and oncogenesis may be aided by an aberrant manifestation of miRNAs [38]. Yang et al. [43] in their study reported that eight miRNAs were dysregulated in HCC, particularly in their phase three study. Additionally, the study utilized four lncRNAs, namely, miR-20a-5p, 320a, 324-3p, and -375, as preclinical biomarkers for HCC diagnosis. Similar studies on miR-106b and miRNAs 21 and 199-a have also been reported to have high diagnostic values for HCC, especially early HCC. Before being used clinically, further validation of the accuracy of miRNAs is needed [42]. Serum levels of miRNA-21 have displayed promising results in differentiating cirrhosis from HCC in small phase II studies. There are several other additional miRNAs that are being studied individually or in combined form in miRNA panels. There are a few challenges with miRNA analyses; however, continuous efforts are underway to assure uniformity in the characterization of miRNA molecules [38]. lncRNAs usually have more than 200 nucleotides, demonstrating diagnostic values for HCC. Research by Li et al. showed that lncRNA HULC and Linc00152 when used in combination have a good diagnosing capacity for the oncogenesis and development of metastasis and could act as novel biomarkers for HCC [44].

Circulating tumor cells (CTCs) are the “seeds” of tumors that travel from cancer cells to the peripheral blood or lymphatic drainage [45]. This process occurs throughout the entire tumor development period. Therefore, CTCs could serve as a good candidate for HCC detection [42]. A study by Guo et al. also demonstrated the clinical implication of CTC in HCC diagnosis using a qPCR-based detection technique [46]. CTCs have the potential to cause metastases in distal organs, which could significantly affect prognosis. Qi et al. in their study showed that the percentages of mesenchymal-CTC were strongly linked to early recurrence, multiple intrahepatic recurrence, and lung metastasis [47].

Circulating cell-free DNA (cfDNA) is an extracellular DNA, released from the tumor cells that are undergoing metabolic secretion, and apoptosis/necrosis. It is like the carrier of tumor-specific genetic or epigenetic changes, DNA methylation, gene alterations, variation, etc. A study on ctDNA which was harvested from the patient’s blood demonstrated genetic and epigenetic changes related to specific types of cancer and their metastatic status. These results can be used for the continuous monitoring of tumor genomes in a non-invasive and precise way. Researchers have been able to examine microsatellite alterations in HCC, such as deletions of chromosomes 17p, 8p, and 19p, with the help of comparative genomic hybridization technologies [48]. A study conducted among Gambian HCC patients showed that Ser-249 protein 53 mutation is one of the most common hotspots [49]. Recently conducted investigations found high recurring hotspot mutations for HCC which could be thought of as potential indicators for the diagnosis of HCC. Droplet digital PCR and high-end sequencing techniques have accurately detected rare mutations in circulating DNA [15].

### Protein Biomarkers

Protein biomarkers are under continuous investigation as there are easier to measure and are present abundantly in the affected body serum. Glycosylation of the proteins is frequently altered during malignant transformation, even in liver cancer. Existing studies on biomarkers need further external validation before using them in clinical settings [50]. The development in proteomics has shed the light on many possible candidates for protein biomarkers. Various biomarkers that have been constantly explored for application in current clinical practice are included in Table 1.

**Table 1:** Overview of protein biomarkers for the diagnostic utility of HCC

Biomarker	Patients	Cut off	AUC (%)	Sensitivity (%)	Specificity (%)	Method
Serum paraoxonase 1 [51]	754	191.12 ng/mL	75.4	70.67	78.11	ELISA
Cyclase-associated protein 2 [52]	86	8.24 ng/mL	81.0	78.6	81.4	ELISA
GP73 [53]	84	8.5 RU	88	73.6	81.5	Immunoblotting
CK19 [54]	102	6.25 ng/mL	NA	63.4	55	ELISA
CCT3 [55]	85	46.5 pg/mL	76.1	76.6	70.5	ELISA
IQGAP3 [55]	85	43.5 pg/mL	75.3	74.5	71.6	ELISA
Thioredoxin [56]	26	20.5 ng/mL	94.6	84.3	91.8	ELISA
Angiopoietin-like protein 2 [57]	361	59.10 ng/mL	83.1	68.25	87.34	ELISA
Dickkopf-1 [58]	370	1.01 ng/mL	82.9	90.7	62.0	ELISA
AKR1B10 [59]	1244	267.9 pg/mL	89.6	72.7	95.7	Time-resolved fluorescent kit
sAxl [60]	240	1202 pg/mL	88.8	95.0	73.3	ELISA
Osteopontin [61]	80	> 19.55 ng/mL	85.3	85.5	72.9	ELISA
Minichromosome maintenance complex component 6 (MCM6) [62]	105	15.50 ng/mL	84.1	67.2	89.8	ELISA
Annexin A2 [63]	90	18 ng/mL	87.3	74	88	ELISA
Human cervical cancer oncogene 1 [64]	570	15 μg/mL	NA	78.2	45.8	ELISA
Glutamine synthetase [65]	260	1.9 mg/mL	91.8	82.9	98.0	ELISA
IgG-L3% [66]	90	24.5%	79.5	86.6	77.7	ELISA
Anti-Ku86 [67]	74	NA	95.4	94	80	ELISA

## Gut Microbiome Biomarkers

The host’s state of health is directly linked to the symbiotic microbial flora of their body. Through the microbiota-liver axis, gut microbial change is probably responsible for the formation of liver cancer as well as the progression of liver disease. It initiates tumorigenesis by integrating the carcinogenic genes in the genome of the host. It affects the stability of the host genome and inhibits the host immune system by breaching the balance between the host immune systems [68]. These characteristics make certain microorganism in the gut a potential marker of HCC prediction.

A large cohort research study that included 419 individuals analysed 16S rRNA MiSeq of fecal samples. The study revealed 30 microbial markers as possible candidates for early HCC detection. This study from China also noticed that phylum *Actinobacteria* and 13 various genera enriched in HCC (initial stage) versus cirrhosis, while microbes of certain genus (butyrate-producers) were decreased in early HCC versus controls. Furthermore, the study identified high prevalence of lipopolysaccharide-producing bacteria *Klebsiella* and *Haemophilus *in HCC patients, and in contrast, *Coprococcus*, *Faecalibacterium*, *Oscillibacter*, *Clostridium* IV, and *Ruminococcus* were depleted in patients with HCC [69]. A study by Yamada et al. [70] and Li et al. [71] reported that microbes *Bacteroides*, *Prevotella*, *Clostridium* XVIII, and *Oscillibacter* were amplified in the HCC patient group compared to a control group, while *Streptococcus*, *Prevotella*, and *Bifidobacterium* were diminished. In clinical trials, results of the study by Grat et al. [72] showed increased *E. coli* levels among HCC patients. On the other hand, Liu et al. [73] reported a decreased level of *Ruminiclostridium*, *Ruminococcus*, *Pseudobutyrivibrio*, *Faecalibacterium*, *Lachnoclostridium*, and *Phascolarctobacterium*. Large-scale research will be necessary for the future to assess the gut microbiome’s potential as a predictor of HCC risk [42].

## Omics, Artificial Intelligence, and Liquid Biopsy: the Future of HCC Diagnosis

Multi-omics-based technology and artificial intelligence (AI) have demonstrated an impact in meeting a critical need for the diagnosis and prognosis of HCC. Several biomarkers are validated in clinical trials. Genomic and epigenetic changes of cfRNA, ctDNA, EVs (extracellular vesicles), and other subsets of cfDNA molecules are all novel biomarkers for HCC [74].

Liquid biopsy is a novel technique adopted in cancer diagnosis in which circulating tumor cells, EVs, nucleic acids, and other biofluids are processed and subjected to molecular analyses. The scope of liquid biopsy extends beyond ctDNA’s genetic and epigenetic modifications. Even the fragment of DNA molecules in circulation provides useful information. A trial conducted by Melter et al. found that exosomes miR-141-3p and miR-375 were pointedly higher in the liver metastasis patient group compared to the control group [75]. In 2021, a method termed the Safe-sequencing system (unique molecular identifier approach to detect rare variants) was reported to have a limit of detection below 0.001% [76]. The fact that all these studies had symptomatic patients could be the greatest limitation, and hence, future research should be planned by aiming toward patients without symptoms.

In the present decade, there is massive growth in the application of AI in medicine, notably in the field of hepatology. Machine learning algorithms are capable of processing a variety of data from healthcare settings’ numeric data, medical documentation, data from multi-omics, and radiological high-resolution images and histopathologic. Convolutional neural networks and deep learning techniques have transformed computer vision and image processing. They can be used to scan images of patients with or without the presence of hepatic lesions using ultrasound, CT, and MRI, sometimes surpassing human radiologists [27].

## Conclusion

Despite a growing research on the oncogenesis of HCC, which allows helps in for the early identification and subsequent management of HCC. There is still a need to evaluate novel biomarkers that are specific to liver malignancies and ensure higher detectability and patient survival. Our review concludes that simultaneous utilization of two or more biomarkers along with omics-based technology and AI might aid in better disease diagnois and management.
